# Research progress of SIRTs activator resveratrol and its derivatives in autoimmune diseases

**DOI:** 10.3389/fimmu.2024.1390907

**Published:** 2024-06-19

**Authors:** Xiaolong Yu, Mingkai Chen, Jiabiao Wu, Ruixiao Song

**Affiliations:** ^1^ Jiangsu Key Laboratory of Immunity and Metabolism, Xuzhou Medical University, Xuzhou, Jiangsu, China; ^2^ Wujin Hospital Affiliated with Jiangsu University, Changzhou, Jiangsu, China; ^3^ The Wujin Clinical College of Xuzhou Medical University, Changzhou, Jiangsu, China

**Keywords:** resveratrol, derivative, SIRTs, sirtuins, autoimmune diseases, SIRTs activators

## Abstract

Autoimmune diseases (AID) have emerged as prominent contributors to disability and mortality worldwide, characterized by intricate pathogenic mechanisms involving genetic, environmental, and autoimmune factors. In response to this challenge, a growing body of research in recent years has delved into genetic modifications, yielding valuable insights into AID prevention and treatment. Sirtuins (SIRTs) constitute a class of NAD-dependent histone deacetylases that orchestrate deacetylation processes, wielding significant regulatory influence over cellular metabolism, oxidative stress, immune response, apoptosis, and aging through epigenetic modifications. Resveratrol, the pioneering activator of the SIRTs family, and its derivatives have captured global scholarly interest. In the context of AID, these compounds hold promise for therapeutic intervention by modulating the SIRTs pathway, impacting immune cell functionality, suppressing the release of inflammatory mediators, and mitigating tissue damage. This review endeavors to explore the potential of resveratrol and its derivatives in AID treatment, elucidating their mechanisms of action and providing a comprehensive analysis of current research advancements and obstacles. Through a thorough examination of existing literature, our objective is to advocate for the utilization of resveratrol and its derivatives in AID treatment while offering crucial insights for the formulation of innovative therapeutic approaches.

## 1 Introduction

Autoimmune diseases (AID) represent a formidable threat to human health, stemming from the immune system’s erroneous targeting of the body’s own tissues. Recent years have witnessed a steady rise in AID incidence, underscoring its status as a critical global public health concern. AID presents with a spectrum of clinical phenotypes due to the production of specific immune cells and autoantibodies during inflammatory immune responses. These assaults on tissues or organs vary widely in their severity (1). Depending on the extent of tissue damage, AID is broadly classified into two principal categories: systemic autoimmune diseases, such as systemic lupus erythematosus (SLE), rheumatoid arthritis (RA), systemic sclerosis (SSc), among others; and organ-specific autoimmune diseases, exemplified by Graves disease (GD), type 1 diabetes mellitus (T1DM), inflammatory bowel disease (IBD), pulmonary fibrosis (PF), and others. The clinical ramifications of AID are profound; for instance, SLE can afflict multiple organs and bodily systems, with severe cases precipitating lupus nephritis, lupus cerebritis, and other crises, posing life-threatening risks (2). RA is characterized by joint degradation, resulting in significant rates of disability (3). Severe manifestations of GD can precipitate cardiovascular irregularities and thyroid crises (4). Individuals with T1DM confront heightened risks of complications such as cardiovascular ailments, retinopathy, and neuropathy (5). Overall, AID is marked by a complex etiology, diverse clinical presentations, and myriad sequelae, exerting considerable impact on individuals’ quality of life and productivity, and imposing substantial economic and psychological burdens on society and families. Presently, the treatment landscape for autoimmune diseases predominantly relies on immunosuppressive agents and biologics; however, these modalities often entail notable side effects, efficacy limitations, considerable interindividual variability, and an inability to effectuate cures. Consequently, the pursuit of precise and efficacious treatment modalities has emerged as a paramount objective in AID research.

## 2 Pathophysiological characteristics of autoimmune diseases

In normal circumstances, the immune system can recognize and respond to external threats, including pathogens and damaged tissues, thereby maintaining the body’s health. However, when the immune system receives abnormal stimuli or its regulation is imbalanced, it can lead to excessive activation, resulting in inappropriate immune responses. It is noteworthy that this abnormal activation can manifest in various forms, such as the overactivation of immune cells, attacks on self-antigens, inflammatory reactions, and more. Additionally, genetic and environmental factors play crucial roles in the abnormal activation of the immune system. The primary mechanisms of immune system abnormal activation include several aspects.

Firstly, the immune system incorrectly identifies its own tissues or molecules as foreign pathogens, which is related to changes in the expression pattern of self-antigens or immune tolerance disorders. Immune tolerance disorder refers to the disruption of immune balance through central and peripheral immune tolerance disorders and abnormal presentation of self-antigens, causing the immune system to mistakenly perceive its own tissues as external threats, resulting in damage to self-tissues (6). Secondly, the abnormal function of immune regulatory cells (such as regulatory T cells) fails to effectively suppress excessive immune responses, leading to the occurrence of autoimmune diseases. Abnormalities in the number and function of regulatory T (Treg) cells, the inhibition of Treg cells by effector T cells, and the increase in abnormal T cell activity can all lead to abnormal activation of the autoimmune system (7). Among them, the balance between Treg cells and T helper 17 (Th17) cells is crucial, as it can restrain the malignant cycle of autoimmunity and block the pathways leading to autoimmune diseases. For instance, the impairment of Treg cells’ stability and the abnormal proliferation of Th17 cells can lead to the activation of other immune cells, thereby driving acute autoimmune responses (8). Research has demonstrated that inhibiting the activity of IL-2 inducible T-cell kinase (ITK) with specific inhibitors can regulate the translocation of Foxo1 and effectively modulate the balance between Th17 and Treg cells (9). This pathway inhibits the phosphorylation process of phospholipase C-gamma 1 (PLC-γ1), mitigating autoimmune reactions and diminishing the production of antigen-specific antibodies. Furthermore, evidence suggests that in the presence of antigen-presenting cells, CD4+ lymphocytes are prompted to activate RORgt via the JAK2/STAT3 pathway, leading to their differentiation into Th17 cells (10). Building on this, research conducted by Wang et al. indicates that obstructing the leptin pathway not only prevents the differentiation of Th17 cells but also enhances the transcription of FOXP3, fostering the conversion of CD4+ lymphocytes into Treg cells (11). Consequently, inhibiting the leptin pathway reinstates the equilibrium between Treg and Th17 cells, thereby reducing cell death attributed to autoimmune inflammation. Thirdly, when the immune system is abnormally activated, inflammatory mediators are excessively released, triggering immune inflammatory reactions. These inflammatory mediators, while regulating and transmitting immune signals, can also cause tissue inflammation and damage. Tumor necrosis factor-alpha (TNF-α) is involved in the inflammatory processes of various autoimmune diseases such as RA and Crohn’s disease (12). Interleukin-1 (IL-1) can cause fever and promote the production of other inflammatory mediators, and is associated with SLE (13). Transforming growth factor-beta (TGF-β) plays a crucial role in controlling immune cell activity and maintaining immune tolerance, but in some cases, it may also promote disease development (14).Fourthly, in Zhernakova’s 2009 whole-genome study, some single nucleotide polymorphisms (SNPs) were found to share and have unique pathological pathways in the development of autoimmune diseases (15). Among them, the most prominent findings were within the MHC locus, which carries multiple signaling pathways from classical pathways of MHCI/II genes to non-classical pathways of MHCIII genes. Within the MHCIII region, multiple genes are involved, including genes encoding complement factor 4 (C4A) and tumor necrosis factor (TNF) (16). In SLE, the HLA-DR3 allele is associated with the production of anti-DNA antibodies (17), while in Sjogren’s syndrome, HLA-DRB1 is associated with the production of Ro antibodies (18). Therefore, the strong association between autoimmune diseases, specific autoantibodies, and specific MHC alleles plays an important role in the occurrence of autoimmune diseases. Fifthly, environmental factors such as infections, drugs, radiation, chemicals, etc., may trigger AID by affecting the function of the immune system or inducing the expression of self-antigens. Smoking is an important risk factor for diseases such as SLE, GD, idiopathic inflammatory myopathy, and IBD (19), and it can promote disease development through various pathways. Cigarette smoke activates innate immune responses and contains Toll-like receptor (TLR) stimulating compounds, directly activating TLR4 signaling and triggering the pro-inflammatory pathway of TLR4 agonists (20, 21). Signals are transmitted through molecules with genetic polymorphisms associated with systemic immunity, providing a direct connection and mechanistic basis for gene-environment interactions. Therefore, specific risk factors will increase the disease risk of individuals with specific genetic backgrounds. Of course, chemicals and drugs such as thiazides, hydralazine, calcium channel blockers, proton pump inhibitors, and interferon-alpha can also induce AID through various mechanisms, including inhibition of central or peripheral tolerance, changes in gene transcription in T and B cells, abnormal cytokine or cytokine receptor function, chromatin structure modification, and antigen modification (22).

## 3 The important role of SIRTs in autoimmune diseases

In 1979, Klar discovered, through mapping studies of the brewing yeast genome, that Sirtuins are mammalian homologs of the yeast silent information regulator 2 (Sir2), thus ushering in the era of research on the SIRTs family (23). Subsequent research indicated that Sirtuins are a class III histone deacetylase dependent on NAD+, present in both higher vertebrates and unicellular eukaryotes within the kingdom Animalia (24). In 1999, Frye et al. identified five human sirtuins, namely SIRT1, SIRT2, SIRT3, SIRT4, and SIRT5, using the amino acid sequence of Sir2 from brewing yeast as a probe (25). Later, the Frye team used human SIRT4 as a probe for similar identification and ultimately discovered two new human sirtuins (SIRT6 and SIRT7) (26). Thus, all seven members of the SIRTs family have been discovered, gradually entering people’s field of vision.

In recent years, research on genetic epigenetic modifications has continued to deepen. Zentner’s study revealed that histones can undergo various covalent translational modifications, including acetylation, methylation, phosphorylation, ubiquitination, and more (27). Subsequently, proteins are recruited to specific modified histones, serving as docking sites, to regulate all chromatin templating processes, such as replication, transcription, and DNA repair. This gene modification creates an open and accessible chromatin environment at all epigenetic markers on the genome, regulating the transcription and repair of DNA (28). However, in this process of genetic modification, enzymes catalyzing the reactions are crucial. The SIRTs family, as NAD-dependent histone deacetylases, can deacetylate lysine residues on histones, regulating the activity of many transcription factors (29). This leads to a silencing effect on numerous target proteins in epigenetics, such as Forkhead box O class (FoxOs), p53, nuclear factor-kB (NFkB), nuclear factor E2-related factor 2 (Nrf2), hypoxia-inducible factor-1α (HIF-1α), AMP-activated protein kinase (AMPK), b-catenin, peroxisome proliferator-activated receptor gamma coactivator 1-alpha (PCG-1a), proliferator-activated receptor gamma (PPARg), and others (30). Through these regulatory mechanisms, SIRTs participate in physiological processes such as inflammation, oxidative stress, mitochondrial function, immune response, and cell differentiation. Particularly, by modulating various cellular signaling pathways, SIRTs play a crucial role in cellular metabolism and DNA repair, making them a promising therapeutic target. Their role in immune regulation includes modulating both innate and adaptive immunity.

### 3.1 The mechanism of SIRTs regulating innate immunity

Innate immunity represents the body’s initial line of defense against pathogens, characterized by its nonspecific, rapid, and broad response. The innate immune system comprises a range of intrinsic immune cells and molecules present from birth, including natural killer (NK) cells, macrophages, monocytes, dendritic cells, neutrophils, eosinophils, and basophils, among others. Unlike adaptive immunity, innate immunity operates independently of prior pathogen exposure and does not confer immune memory against specific pathogens. It is noteworthy that the SIRTs family can modulate the function of innate immune cells at multiple levels. Generally, decreased sirtuin levels can precipitate various inflammatory states in the body, including autoimmunity, cellular senescence, and degenerative changes in tissues and organs.

#### 3.1.1 SIRTs and macrophages

Macrophages arise from early embryonic erythro-myeloid progenitors or from adult infiltrating monocytes. Upon antigen stimulation, macrophages are activated and polarized into pro-inflammatory or anti-inflammatory phenotypes, namely classical activation (M1) macrophages and alternative activation (M2) macrophages. M1 macrophages execute cytotoxic and tissue-damaging pro-inflammatory functions, while M2 macrophages play a crucial role in resolving inflammation and tissue repair (31). Within the SIRTs family, SIRT1, SIRT2, SIRT6, and SIRT7 participate in multiple specific stimuli and downstream signaling events of macrophage polarization, with significant implications for balancing macrophage polarization. Several studies have shown that deletion of the SIRT1, SIRT2, and SIRT6 genes in macrophages results in a significant increase in acetylation levels of the p65 subunit of NF-kB, leading to increased expression of NF-kB target genes IL-6, TNF-α, and IL-1β, inducing immune inflammation (32, 33). In other words, SIRT1, SIRT2, and SIRT6 inhibit inflammation in macrophages by deacetylating NF-kB (34). Rothgiesser et al. found that SIRT2 interacts with p65 in mouse embryonic fibroblasts, leading to deacetylation of p65 subunit of NF-kB at Lys 310, thereby increasing the expression of p65 acetylation-dependent target gene subsets, regulating the expression of specific NF-κB-dependent genes, and controlling a large number of target genes involved in immune and inflammatory responses, cell proliferation, differentiation, and apoptosis (35). Santos-Barriopedro et al. found that SIRT6 can bind to the histone methyltransferase Suv39h1 specific for H3K9me3 and induce monoubiquitination at the conserved cysteine residue in the PRE-SET domain of Suv39h1 (36). Upon activation of the NF-κB signaling pathway, SIRT6 upregulates the activity of the NF-κB inhibitor (IκBα) through cysteine monoubiquitination and chromatin eviction by Suv39h1, thus achieving inhibition of the NF-κB pathway and attenuating the aggravation of the immune-inflammatory response by SIRT6. In addition, the expression of SIRT7 decreases in an age-dependent manner in leukocytes of healthy individuals, and Phorbol-12-myristate-13-acetate(PMA)-mediated monocyte-to-macrophage differentiation in the monocytic THP-1 cell line increases SIRT7 expression, while SIRT7 overexpression also increases differentiation of non-stimulated THP-1 cells (37).

#### 3.1.2 SIRTs and NK cells

NK cells are cytotoxic lymphocytes that play a crucial role in innate immunity against viral infections and tumors. NK cells secrete perforins and granzymes, inducing apoptosis in target cells through the expression of cell death ligands on their surface. Additionally, NK cells secrete various pro-inflammatory cytokines, including TNF-α and interferon-beta (IFN-β), which play important roles in maintaining and amplifying immune responses through interactions with macrophages and dendritic cells (38). Studies have indicated a specific increase in the expression of SIRT2 in liver NK cells induced in mice with liver cancer, suggesting that SIRT2 promotes the activity of liver NK cells in response to hepatocellular carcinoma (HCC). Furthermore, overexpression of SIRT2 enhances the secretion of pro-inflammatory cytokines and cytotoxic granules by NK cells, thereby exhibiting increased anti-tumor activity (39). Additionally, SIRT2 activity is associated with increased phosphorylation of extracellular signal-regulated kinases 1/2 (Erk1/2) and p38 mitogen-activated protein kinase (MAPK), which are two important signaling pathways for NK cell activity (40). Therefore, SIRT2 holds potential value in enhancing the anti-tumor effects mediated by liver NK cells.

#### 3.1.3 SIRTs and dendritic cells

Dendritic cells are antigen-presenting cells that, under steady-state conditions, exhibit high phagocytic activity and continuously present self-antigens to restrain T cell reactivity. Upon infection, dendritic cells mature, leading to an increased expression of co-stimulatory receptors, including CD80, CD86, and MHC-II molecules (41). When dendritic cells show weakened responses to pathogens but enhanced responses to self-antigens, it results in an increased expression of pro-inflammatory cytokines, leading to the breakdown of immune tolerance and inflammatory responses.

Research indicates that the expression of SIRT1 in dendritic cells increases when toll-like receptors (TLRs) are stimulated. Conversely, the absence of SIRT1 leads to changes in T cell polarization (42). In 2012, Alvarez et al. demonstrated that SIRT1 promotes the production of the signature cytokines IL-12 and TGF-β1 in a hypoxia-inducible factor α (HIF1α)-dependent manner, thereby regulating the differentiation of Th1 cells and Treg cells, participating in the process of immune tolerance regulation (43). Subsequently, Yang et al. found that in dendritic cells, SIRT1 interacts with interferon regulatory factor 1 (IRF1), a transcription factor associated with the expression of interleukin 27 (IL-27), and produces a deacetylation effect. This effect reduces the binding of IRF1 to the gene promoter of il-27p28, silencing it and decreasing the production of IL-27, promoting the differentiation of Th17 cells, which are a pro-inflammatory subset of T cells (44). In summary, SIRT1 is a major regulatory factor for cytokine production in dendritic cells and holds significant importance for the subsequent generation of T cell subsets. Apart from SIRT1, SIRT6 also participates in the differentiation and maturation of dendritic cells. Studies confirm that inhibiting SIRT6 significantly hinders the differentiation of monocytes into dendritic cells, resulting in immature dendritic cells with significantly reduced expression of CD86, CD80, and MHC-II molecules. Additionally, these dendritic cells exhibit increased phagocytic ability and a further decrease in the ability to stimulate lymphocyte proliferation (45, 46). At the same time, there is an increased proportion of cells producing TNF-α and IL-6, indicating that SIRT6 regulates the production of cytokines in these cells.

### 3.2 Mechanisms of SIRTs regulation in adaptive immunity

Adaptive immunity constitutes the highly specific and memory-driven immune response of an organism against specific pathogens. In contrast to innate immunity, which broadly recognizes nonspecific antigens present in pathogens and damaged host cells, the adaptive immune system possesses high antigen recognition specificity, immunological memory, and adaptability to diverse pathogens. Primarily composed of T cells and B cells, the adaptive immune system features B cell receptors (BCRs) or T cell receptors (TCRs) expressed on their cell membranes. While tolerating self-antigens, these cells have the capability to recognize and mount highly specific immune responses against particular antigens.

#### 3.2.1 SIRTs and T lymphocytes

T lymphocytes comprise helper CD4+ T cells and cytotoxic CD8+ T cells, activated by TCR-specific antigens during cell-cell contact. Fagnoni et al. (47) propose an association between SIRT1 and the accumulation of CD8+CD28- T cells. Effros et al. (48) discovered that under conditions of CD28 co-stimulation deficiency, CD8+CD28- T cells exhibit heightened cytotoxicity, express pro-inflammatory cytokines, and display characteristics indicative of replicative senescence. During aging, SIRT1 undergoes autophagy-mediated degradation in various organs, including the spleen and thymus. Jeng and colleagues observed a notable decrease in SIRT1 levels in human CD8+ memory T cells, particularly CD8+CD28- T cells, without alterations in their gene expression. SIRT1, is a deacetylase for many transcription factors, including FOXO1, and regulates several cellular processes, such as proliferation, differentiation, and apoptosis. SIRT1 activation triggers apoptosis by directly inhibiting FOXO1 expression through deacetylation. Consequently, the loss of SIRT1 amplifies proteasomal degradation of its target FOXO1, thereby diminishing the cytotoxicity of memory T cells by enhancing their glycolytic capacity (49). Studies indicate a substantial decrease in SIRT2 levels in the spleens of aged rats, with an even more precipitous decline in SIRT2 levels in the thymus. Moreover, Sidler et al. (50) found that among the seven sirtuin genes encoded by the mammalian genome, only the expression level of SIRT2 decreases with age. This may be linked to the relationship between SIRT2 and Histone H4K16, a target of SIRT2 deacetylation in aging yeast (51). Reduced DNA methylation and H3K9me3 with age suggest the loss of heterochromatin as aging progresses. Additionally, SIRT6 plays a role in immune aging and inflammatory responses by regulating T cell inflammatory responses. A study of SIRT6 in aging demonstrated that targeted deletion of SIRT6 in T cells or the myeloid lineage recapitulated the inflammatory and fibrotic phenotype in the liver, suggesting autonomous regulation of inflammation by SIRT6 in immune cells (52). Importantly, SIRT1 levels are significantly upregulated in patients with GD, promoting the deacetylation of FOXP3 and mediating the simultaneous loss of IL-17+ T cells and Treg cells in Graves’ patients (53). Overexpression of SIRT1 in patients with IBD induces concurrent inhibition of Th17 cell differentiation and Treg cell differentiation, exacerbating colitis development. In conclusion, SIRTs actively participate in T cell differentiation processes, influencing immune responses and contributing to the occurrence of autoimmune diseases.

#### 3.2.2 SIRTs and B lymphocytes

B lymphocytes constitute the cornerstone of adaptive immunity. Mature B cells predominantly reside in the spleen and lymph nodes, displaying antigen-specific immunoglobulins on their cell membranes. Upon infection, antigen-specific B cell clones are activated, leading to their differentiation into plasma cells that secrete antibodies or memory B cells.

The intrinsic relationship between SIRTs and B lymphocytes is currently under exploration. Nonetheless, it is established that SIRTs are linked to immunoglobulin class switch recombination (CSR), B cell homeostasis, and autoimmune suppression. Research indicates a significant role for SIRT7 in non-homologous end joining (NHEJ) repair. Regarding NHEJ, as one of the main DNA double-strand break repair modes in cells. NHEJ is different from homologous recombination in that it does not require homologous DNA as a repair template and can repair broken DNA at all stages of the cell cycle (54). In addition, NHEJ is involved in the recombination of diversified antibodies and T-cell receptors. Reduced expression of SIRT7 during aging may partly contribute to the diminished clonal diversity of newborn B cells, associated with immunosenescence (55). In a study by Gan in 2020, it was discovered that the activation of immunoglobulin CSR is mediated by activation-induced cytidine deaminase, and its expression in mature B cells is subject to epigenetic regulation. Enhanced activity of SIRT1 can lead to the deacetylation of H3K9Ac/H3K14Ac in the promoter of activation-induced cytidine deaminase, resulting in reduced expression and impaired CSR (56). Moreover, SIRT1 is crucial for B cell antigen presentation, as the loss of SIRT1 in B cells leads to decreased levels of MHC-II molecules expressed, thereby reducing their cross-presentation with CD4+ T cells (57). In summary, the downregulation of SIRT1 with age may compromise B cell immunity and contribute to immunosenescence.

Notably, during immunosenescence, inflammation and AID are commonly observed. In mature B cells of SLE patients, significantly lower levels of SIRT1 have been observed, and SIRT1 levels are negatively correlated with the frequency of CD19+ B cells in these patients (58). Activation of SIRT1 has a protective effect on RA patients, partly due to a reduction in the production of autoantibodies by B cells (59). Serum levels of SIRT1 in asthma patients are positively correlated with IgE levels (60), while anti-SIRT1 autoantibodies are more abundant in inflammatory diseases such as ankylosing spondylitis (61). In summary, SIRT1, by inhibiting the autoimmune response of B cells and reducing the cascade of immune inflammation, plays a crucial role in preventing severe invasion of various systems and organs in the body, maintaining the stability of the immune environment, and ensuring the normal functioning of the immune system.

## 4 The development history of resveratrol and its derivatives

Resveratrol (RES) is a natural phytotoxin found in many plants, produced in response to ultraviolet radiation, damage, fungal, or bacterial infections (62). It is present in various natural foods, including grapes, blueberries, and mulberries, with significant variations in concentration among different foods (63). The chemical structure of RES consists of two aromatic rings connected by a methylene bridge, with both trans and cis isomers naturally occurring. The primary medicinal effects of RES stem from the trans-resveratrol, making the structure-activity relationship crucial in determining RES bioactivity (64). However, due to its rapid absorption and metabolism in the human body, RES exhibits low plasma concentrations and tissue distribution (65). Consequently, there has been widespread interest in finding methods to enhance the bioavailability of RES.

RES is a small molecule with a molecular weight of 228.247 g/mol and various functional groups, including phenolic hydroxyl, aromatic rings, and double bonds (66). These functional groups provide opportunities to modify RES into active derivatives with diversified therapeutic effects. Therefore, designing and synthesizing novel resveratrol derivatives to enhance the therapeutic effects of RES has become a hotspot of interest among pharmacologists. Over the past few decades, research on various natural and synthetic resveratrol derivatives, particularly methoxylation, hydroxylation, and halogenation derivatives, has received special attention. Here, we provide a comprehensive summary of the chemical structures, mechanisms of action, and clinical effects of methoxylated, hydroxylated, and halogenated derivatives of RES (**Table 1**).

**Table 1:** Chemical structure, mechanism of action and clinical effects of resveratrol derivatives.

Classification	Name	Chemical structure	Differences with resveratrol	Mechanism of action	Clinical effect	References
**Methoxylated resveratrol derivatives**	Pterostilbene	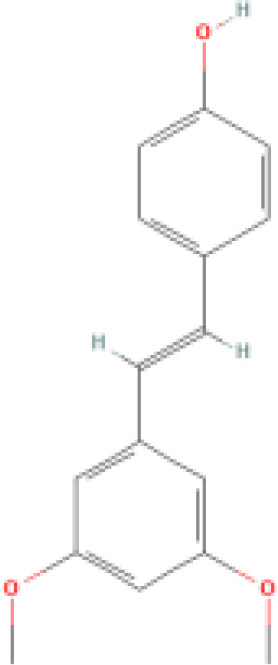	More lipophilic than resveratrol.	1. Inhibits various signaling pathways. 2. Suppresses ethanol-induced DNA oxidative damage.	1. Inhibits oxidative stress. 2. Reduces the chemosensitivity of pancreatic cancer, melanoma, leukemia, breast cancer, lung cancer, and gastric cancer. 3. Promotes apoptosis. 4. Anti-invasive, anti-metastatic, and anti-inflammatory. 5. Treats hypertension and diabetes.	(67–70)
	Trimethoxystilbene	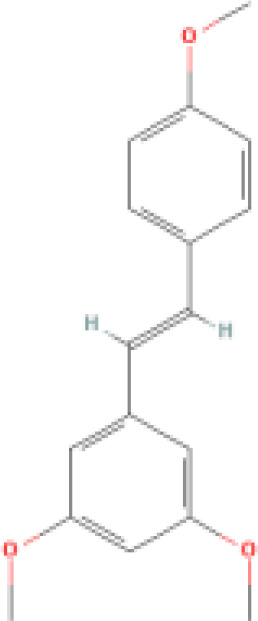	Stronger by 30-100 times in inhibiting endothelial cell proliferation and morphological transformation compared to resveratrol.	1. Downregulates the activity of the PI3K/AKT signaling pathway. 2. Acts as an anti-angiogenic compound to block neovascular formation. 3. As a vascular-targeting agent, it can cause rupture of immature blood vessels. 4. Promotes bundling of microtubules and formation of spot-like structures of DCLK1-microtubule complexes.	1. Used in chemotherapy for breast cancer, lung cancer, and liver cancer. 2. Vascular-targeted therapy. 3. Inhibits HCV.	(71–73)
	Tetramethoxy stilbene	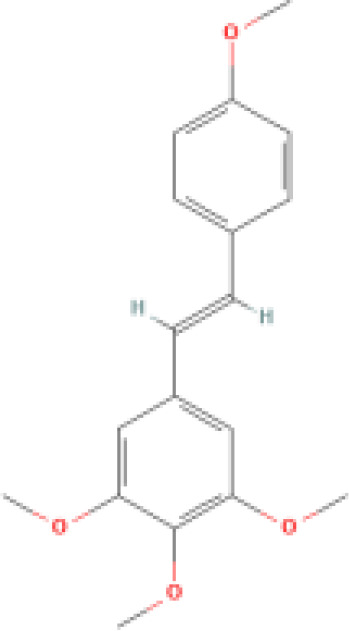	Possesses higher biological potency and bioavailability than resveratrol.	1. Induces the release of a large amount of cytochrome C into the cytoplasm by increasing mitochondrial permeability at the nuclear periphery. 2. Exhibits anti-angiogenic activity by inhibiting the phosphorylation of multiple downstream signaling components of VEGFR2.	1. Inhibits the growth of various cancers, including colon cancer, prostate cancer, ovarian cancer, and liver cancer. 2. Inhibits cytochrome P450. 3. Regulates hypertension and cardiac fibrosis.	(74–77)
	Pentamethoxystilbene	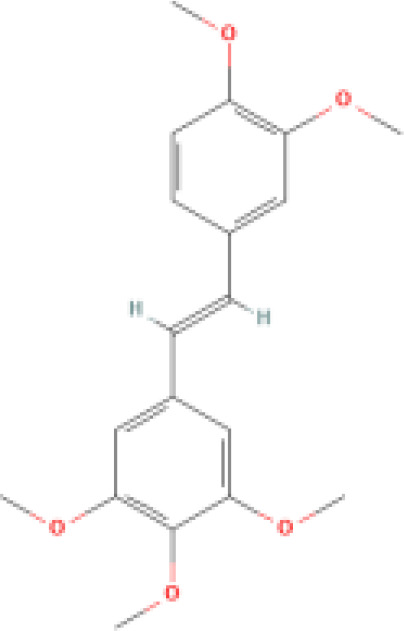	Far exceeds resveratrol in inhibiting cell growth.	Induces cell cycle G1 phase arrest and regulates G1 phase proteins.	1. Inhibits the growth of breast cancer and colon cancer. 2. Inhibits cytochrome P450.	(78, 79)
**Hydroxylated resveratrol derivatives**	Dihydroxystilbenes	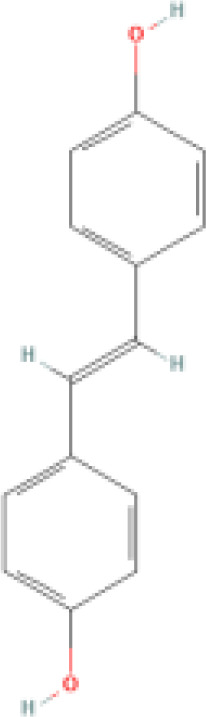	Exhibits stronger biological effects than resveratrol.	1. Inhibits cancer progression by blocking G1 phase cell transition and tumor growth. 2. Reduces the secretion of endothelin-1 and mRNA levels of endothelin-1 in human endothelial cells. 3. Protects against hemin-induced lipid peroxidation and ROS production.	1. Used in chemotherapy for lung cancer and breast cancer. 2. Manages vascular abnormalities. 3. Inhibits oxidative stress reactions.	(80, 81)
	Tetrahydroxystilbene	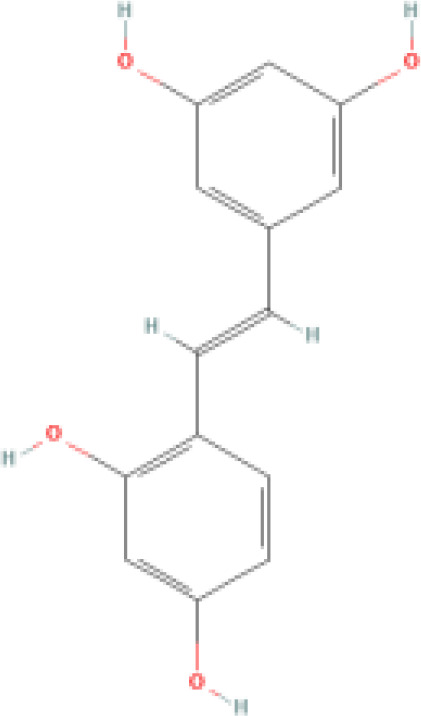	Stronger anti-oxidant and anti-cancer activity than resveratrol.	1. Inhibits oxidation of LDL-C in plasma, platelet aggregation, and inflammation. 2. Antiproliferative, cytotoxic, and inhibitory effects on cancer cell growth. 3. Inhibits mutations in the leucine-rich repeat kinase-2 and uptake of 5-hydroxytryptamine.	1. Regulates atherosclerosis, hypertension, and myocardial ischemia. 2. Reduces the chemosensitivity of liver cancer, leukemia, and cervical cancer. 3. Inhibits cell oxidation. 4. Antibacterial. 5. Effective against Parkinson's and Alzheimer's disease.	(82–84)
	Hexahydroxystilbene	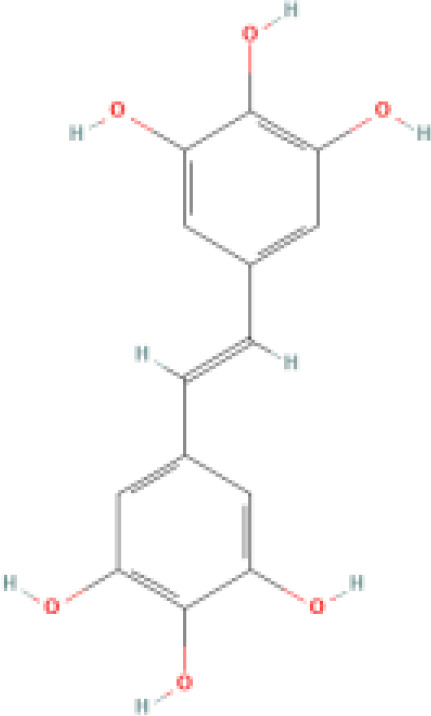	Most effective radical scavenger among resveratrol derivatives, with higher anti-HIV-1 activity than resveratrol.	1. Causes imbalance of deoxyribonucleotide triphosphate pools within cells. 2. Inhibits virus attachment and reverse transcription.	1. Inhibits the growth of various malignant tumors, including breast cancer, colon cancer, leukemia, melanoma, and glioma. 2. Inhibits HIV-1. 3. Selective COX-2 inhibitor. 4. Inhibits oxidative stress.	(85–87)
**Halogenated resveratrol derivatives**	(E)-2,6-dibromo-4-(3,5-dibromostyryl)phenol	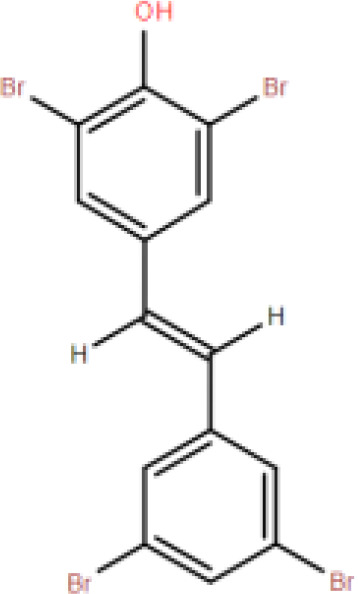	Higher bioavailability than resveratrol.	Stabilizes the natural tetramer of transthyretin and modifies the quaternary structure of monomeric transthyretin in solution.	Cardioprotective effects.	(88)
	(E)-3,5-di-fluoro-4'-acetoxystilbene	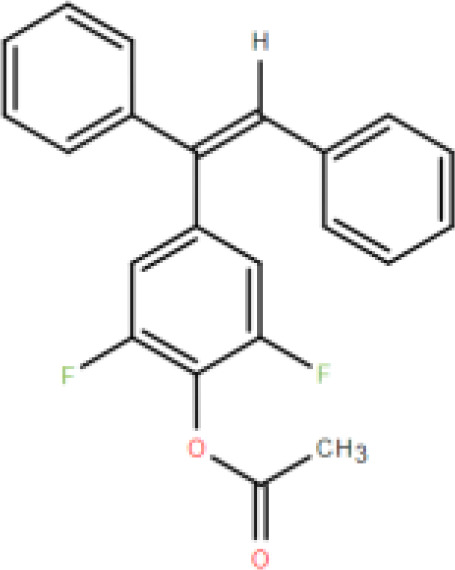	Stronger anti-tumor activity than resveratrol.	Inhibits upregulation of ABC superfamily cell transport proteins.	Resists cell proliferation.	(89)
	3,4,5-trimethoxy-4'-brom-cis-stilbene	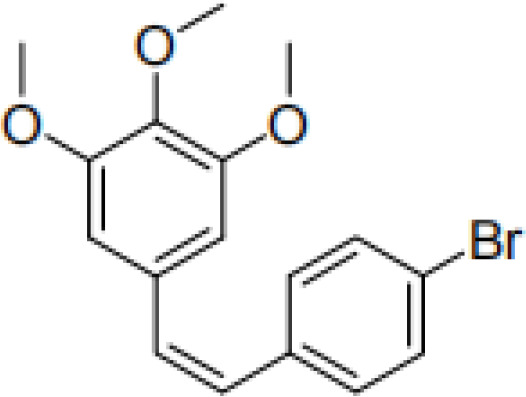	Stronger anti-cancer effects than resveratrol.	Inhibits G2/M phase tumor cell growth.	Inhibits lung cancer cell growth.	(90)
	4'-Bromo-Resveratrol	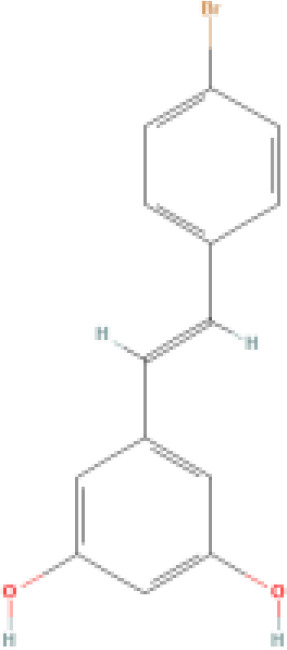	More effective in inhibiting SIRT3 than resveratrol.	Effectively inhibits SIRT1 and SIRT3 by extending the bromophenyl moiety of the active site.	Therapeutic effects on aging, transcription, apoptosis, and inflammation-related diseases.	(91)
	2-bromo-resveratrol	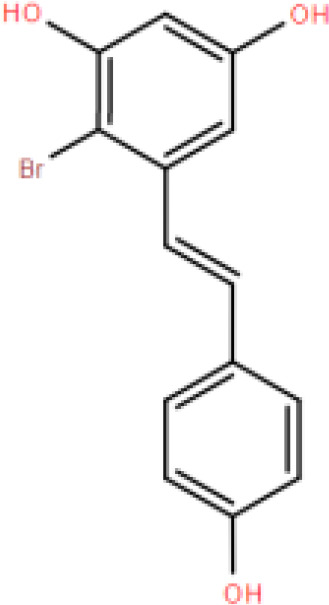	MIC values against Candida albicans are 3 times lower than resveratrol.	—	1. Inhibits microbial growth. 2. Inhibits cell proliferation.	(92)
	2-chloro-resveratrol	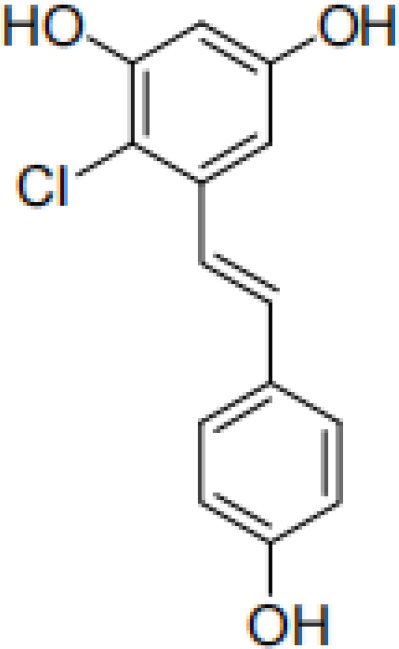	MIC values against Candida albicans are 30 times lower than resveratrol.	—	1. Inhibits microbial growth. 2. Inhibits cell proliferation.	(92)

In 2003, David Sinclair and colleagues utilized the innovative “FluordeLys” (FdL) method and discovered the first-generation SIRT1 activator – RES (93), also the first SIRTs activator discovered in humans. As a pioneer of SIRTs activators, RES has rich clinical applications and promising therapeutic prospects.

Combretastatin A-4, a cis-configured methylated resveratrol derivative, was first isolated from Combretum caffrum in 1995. However, it gained prominence in 2003 due to its phosphate salt, which selectively disrupts the microtubule protein cytoskeleton of endothelial cells (94), exhibiting potent anti-tumor and anti-vascular effects, leading to the development of the market drug ombrabulin (95).

In 2004, Rimando and colleagues found that Pterostilbene (96), structurally highly similar to RES, also possesses potent anti-tumor, anti-inflammatory, and antioxidant activities (97), serving as a naturally occurring dimethylated resveratrol derivative from blueberries. The dimethoxy structure of Pterostilbene enhances its lipophilicity and cellular membrane permeability, with better metabolic stability than RES, displaying superior pharmacokinetic properties (98). Research indicates that Pterostilbene can more effectively prevent azoxymethane (AOM)-induced colon cancer by activating the antioxidant signaling pathway mediated by NF-E2-related factor 2 (Nrf2) compared to RES (99).

Piceid, a non-glycosylated resveratrol derivative, is the most abundant form of RES in nature (100). It is primarily found in alcohol-free grape juice and is also a major extract component of the traditional herbal medicine Polygonum cuspidatum, used to treat heart diseases, including atherosclerosis and myocarditis (101). Jeong et al. discovered in 2010 that Piceid inhibits tyrosinase in melanocytes, thus blocking melanin production without adversely affecting cell viability. Additionally, Piceid inhibits tyrosinase, tyrosinase-related protein 1 (TRP1), tyrosinase-related protein 2 (TRP2) and microphthalmia-associated transcription factor (MITF), leading to reduced melanin synthesis (102). Hence, Piceid may be a potential candidate for a skin whitening agent. Recent studies show that the team led by Kobayashi et al. isolated a resveratrol glucosyltransferase gene from three grapevine species and introduced it into kiwi plants via Agrobacterium-mediated gene transfer, producing the glycosylated form of RES – piceid (103).

In 2011, Hsieh et al. observed in prostate cancer cells LNCaP that resveratrol derivatives triacetyl-resveratrol and trimethoxy-resveratrol are effective anti-CaP agents (104). Due to their evasion of further enzymatic glucuronidation or sulfation, they exhibit efficacy comparable to RES in chemoprevention and treatment. Furthermore, triacetyl-resveratrol preferentially targets the inhibition of CaP proliferation through the p53 signaling pathway, while trimethoxy-resveratrol acts by controlling the cell cycle and inducing apoptosis, achieving a dual anti-CaP activity (105). As the most extensively studied and structurally simplest resveratrol derivatives to date, tri-methylation and tri-acetylation resveratrol derivatives play indispensable roles in regulating the growth and gene expression of prostate cancer cells LNCaP (106).

Pallidol is one of the most common resveratrol oligomers, relatively easy to obtain. It effectively scavenges reactive oxygen species (ROS) and activates the Keap1-Nrf2 pathway, upregulating the expression of antioxidant enzymes (107). In 2015, the team led by Matsuura B developed an efficient and scalable oxidative dimerization method for the tert-butylated resveratrol derivative, resulting in the unique and stable quinone methylation product pallidol, the most effective synthesis method of pallidol to date (108).

Duan et al. designed and synthesized a series of resveratrol derivatives in 2016, proven to be effective lysine-specific demethylase 1 (LSD1) inhibitors (109). Among them, the compounds (Z)-3-((E)-2-bromo-4,5-dihydroxystyryl)-N’-hydroxybenzimidamide (4e) and (Z)-4-((E)-2-bromo-4,5-dihydroxystyryl)-N’-hydroxybenzimidamide (4m) were identified as the most effective LSD1 inhibitors, dose-dependently inducing an increase in dimethylation of Lys4 of histone H3 in MGC-803 cells, while significantly increasing the mRNA levels of CD86 (an alternative cellular biomarker of LSD1 activity) in MGC-803 cells (110), demonstrating potent intracellular inhibition of LSD1 with potential anticancer activity. Furthermore, inhibiting LSD1 can enhance the binding of histone H3 lysine 4 dimethylation (H3K4me2) to its promoter sequence, upregulating the expression of transferrin receptor (TFRC) and acyl-CoA synthetase long-chain family member 4 (ACSL4). Subsequently, this promotes the accumulation of intracellular iron and the synthesis of unsaturated fatty acids, leading to ferroptosis. Ferroptosis, to some extent, can inhibit tumor growth and metastasis. Therefore, these resveratrol derivatives may become a novel option for cancer therapy.

In 2020, Yang et al. found that Piceatannol (111), a hydroxylated resveratrol derivative, exhibits biological functions similar to RES (112). In MDA-MB-231 breast cancer cells, it inhibits cancer cell invasion, migration, adhesion, and reduces the activity of matrix metalloproteinase-9 (MMP-9), thereby suppressing breast cancer cell proliferation, invasion, and metastasis (111, 113). Meanwhile, Piceatannol reduces drug metabolism rates, increases its bioavailability, and surpasses RES in terms of drug potency by participating in multiple steps such as apoptosis, cell proliferation, and clonogenicity (114).

In 2022, Innets’s research found that the methoxy derivative of RES, 4,4-(ethane-1,2-diyl) bis(2-methoxyphenol) (RD2), exhibits high affinity between the ATP binding site and the allosteric site of the Akt molecule (115). In other words, RD2 is a potential inhibitor of Akt. RD2 blocks the PI3K/Akt/mTOR pathway (116), leading to the activation of apoptosis, reduction in cell proliferation rate, and inhibition of cancer cell metastasis, making it a potential drug for the treatment of non-small cell lung cancer (117).

In 2023, Fragopoulou’s research showed that the methoxy derivatives of RES have better antiplatelet effects, with the most effective derivative being the 4’-methoxy derivative, exhibiting approximately 2.5 orders of magnitude of antiplatelet activity against thrombin receptor activating peptide (TRAP)-induced platelet aggregation, indicating its potential as an antiplatelet agent comparable to protease-activated receptor-1 (PAR1) inhibitor vorapaxar (118).

**Figure 1:**
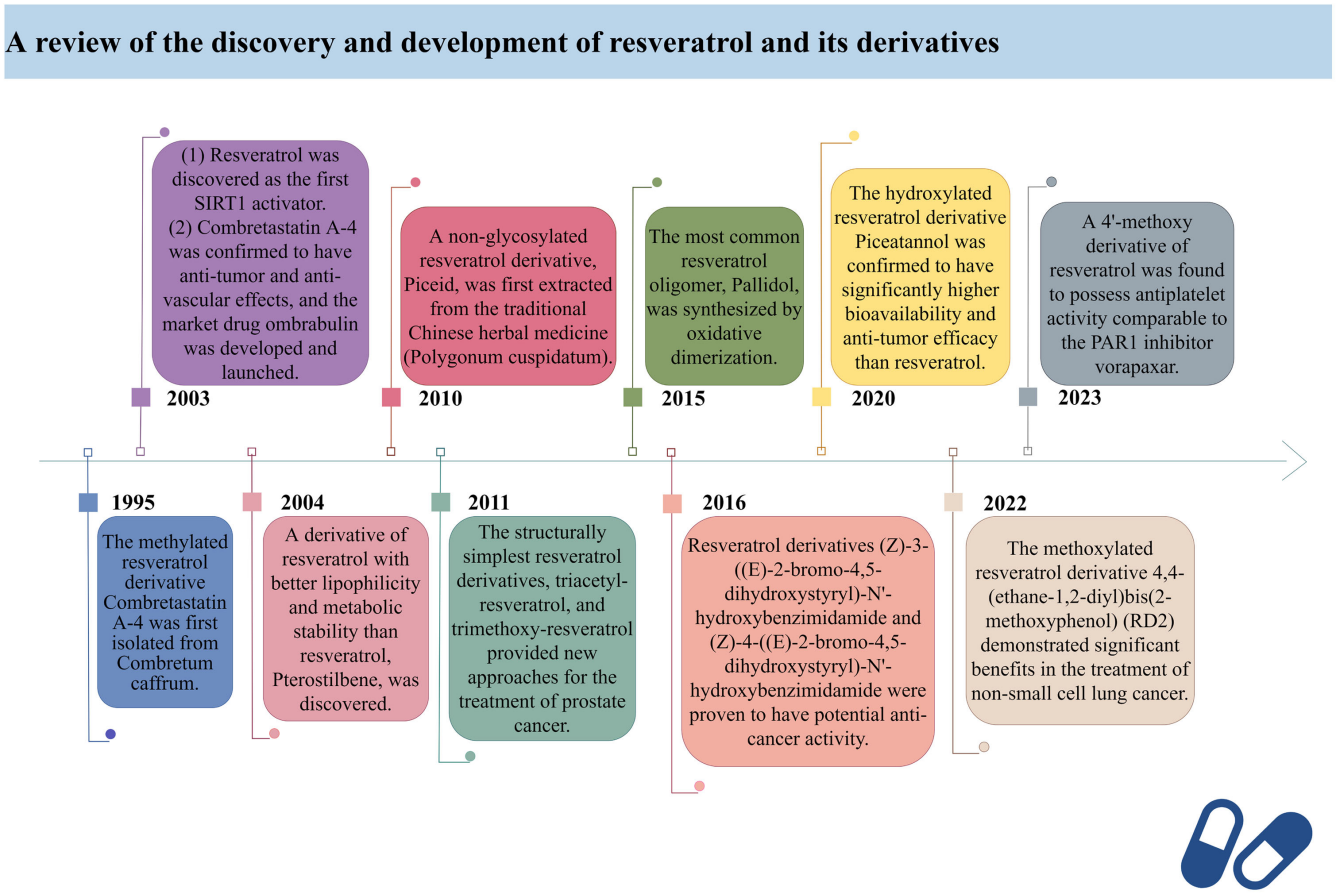
A review of the discovery and development of resveratrol and its derivatives.

Here, we have retrospectively reviewed the discovery timeline and development process of resveratrol and its derivatives, providing a brief overview of their specific efficacy in clinical applications (**Figure 1**).

## 5 Resveratrol and its derivatives in the research progress of autoimmune diseases

### 5.1 Systemic autoimmune diseases

#### 5.1.1 Rheumatoid arthritis (RA)

RA is a systemic autoimmune disease characterized by erosive joint damage and systemic inflammation, affecting multiple systems (119). It is among the most prevalent systemic autoimmune diseases, with increasing prevalence and disability rates annually, particularly among middle-aged and elderly women aged 30–60, in regions such as North America, Europe, and Asia (120). The hazards of RA primarily involve joint damage and deformity, alongside systemic inflammation and elevated cardiovascular risk, significantly impacting patients’ quality of life and imposing a substantial global economic burden (121–123).

Oxidative stress is widely recognized as a major contributor to inflammation and arthritis. An increase in chemical reactions within the body, damage to antioxidant defense systems, and an imbalance between oxidants and antioxidants can lead to a sharp rise in ROS, culminating in the accumulation of reactive oxygen molecules and defects in DNA, RNA, and proteins (124). Studies have demonstrated that RES effectively scavenges radicals such as hydroxyl and superoxide, thereby inhibiting lipid peroxidation and DNA damage induced by excessive ROS production (125). Consequently, RES exhibits remarkable anti-inflammatory and antioxidant effects in RA. During oxidative stress, nuclear factor erythroid 2-related factor 2 (Nrf2), a transcription factor with antioxidant and anti-inflammatory properties, counteracts mitochondrial decay by interacting with antioxidant response elements (AREs) (126). Building upon this theoretical foundation, Li et al. revealed that RES can prevent calcium deposition and mitochondrial decay by activating the SIRT1/Nrf2 pathway, thereby inhibiting the phosphorylation and acetylation of NF-κB p65 and suppressing synovial hyperplasia and joint inflammation (127).

Given SIRT1’s capability to deacetylate various transcription factors, including NF-κB, it can modulate downstream cellular pathways associated with oxidative stress (128). Consequently, activating SIRT1-mediated NF-κB deacetylation to inhibit oxidative stress and inflammation has emerged as a potential therapeutic strategy for RA. In 2012, Rathore identified that NF-κB p65 directly targets the miR-29a-3p and miR-23a-3p promoters, downregulating the transcription levels of miR-29a-3p and miR-23a-3p, thus inducing immune inflammatory responses and oxidative stress in the body, leading to joint synovial damage (129, 130). Subsequently, Wang’s research in 2019 demonstrated that RES can inhibit the acetylation and phosphorylation of NF-κB p65, increase the transcriptional activity of miR-29a-3p and miR-23a-3p promoters, block fibroblast-like synoviocyte (FLS) proliferation, and mitigate oxidative stress induction mediated by miR-29a-3p and miR-23a-3p promoters, thereby alleviating ROS-induced damage to joints and surrounding tissues (131). In summary, RES regulates the activation of the SIRT1/NF-κB/miR-29a-3p/Keap1 pathway and the SIRT1/NF-κB/miR-23a-3p/cul3 pathway, subsequently activating the Nrf2-ARE signaling pathway, effectively inhibiting oxidative stress resulting from ROS accumulation and mitigating joint inflammation, positioning it as a promising candidate drug for RA prevention and treatment.

Thalhamer et al.’s research highlights the pivotal role of MAPK signaling pathways in RA, particularly emphasizing the significance of p38 MAPK (132, 133). Essentially, ROS trigger inflammation by activating the MAPK signaling pathway, which subsequently induces the production of pro-inflammatory cytokines, perpetuating a detrimental cycle of immune-driven inflammation resulting in severe bone erosion (134). In 2018, Yang et al. demonstrated that the functional inhibition of p38 MAPK by RES underpins its protective effects against RA progression (135). Specifically, RES can activate SIRT3, thereby restoring enzymatic activities of isocitrate dehydrogenase 2 (IDH2), superoxide dismutase 2 (SOD2), and glutathione peroxidase (GSH-Px) in endothelial cells, and promoting the deacetylation of SOD2 and forkhead box O3A (FoxO3A) (136, 137). Through this mechanism, RES diminishes ROS accumulation, inflammation, and angiogenesis in synovial tissue, inhibits p38 MAPK activation, and consequently exerts anti-inflammatory, anti-angiogenic, cell-inhibitory, and pro-apoptotic effects, exhibiting preventive potential against RA both *in vitro* and *in vivo*. Therefore, targeting p38 MAPK represents a promising approach for RA treatment, and RES emerges as a promising candidate for clinical RA therapy.

Khojah and colleagues conducted a randomized controlled clinical trial to investigate the efficacy of RES therapy in treating RA, wherein they found that RES effectively reduces the levels of TNF-α, IL-1β, IL-6, monocyte chemoattractant protein 1, and soluble receptor activator of NF-κB ligand in both serum and joint tissues (138). Subsequent studies indicate that RES achieves this reduction by upregulating SIRT1 expression levels, resulting in a significant decrease in NF-κB expression and thereby mitigating inflammation and bone degradation (139). Furthermore, RES demonstrates regulatory effects on inflammatory arthritis in rodents by selectively inhibiting cellular and humoral responses associated with RA development (59). Additionally, RES induces apoptosis in RA patients’ fibroblast-like synoviocytes (FLS) by activating caspase-8, leading to substantial apoptotic cell death through both mitochondrial signaling pathway convergence and caspase-dependent pathways (140). Consequently, the emergence of RES presents a promising, efficacious, and safe therapeutic option for RA treatment (141).

#### 5.1.2 Systemic lupus erythematosus (SLE)

SLE patients produce autoantibodies such as antinuclear antibodies (ANA) that attack their own tissues and organs, releasing large amounts of inflammatory mediators such as TNF-α and IL-6, thereby triggering immune and inflammatory responses (142). SLE is a globally distributed systemic autoimmune disease involving genetic susceptibility genes and environmental factors such as infections, drugs, and ultraviolet light in its pathogenesis (143). Currently, SLE is widely recognized worldwide due to its complex clinical manifestations and severe complications. Its hazards mainly include lupus nephritis, cardiovascular diseases, central nervous system damage, risks of infection and bleeding, affecting female fertility, and increasing pregnancy risks. Severe cases may lead to systemic multi-organ damage and lupus crisis (144–148).

Research indicates a close relationship between CD4+ T cells and B cells in SLE (149). In humoral immunity, helper T cells interact with B lymphocytes, stimulating their proliferation and differentiation, leading to the production of autoantibodies against nuclear components, thereby attacking self-tissues and organs (150), ultimately triggering systemic autoimmune diseases. However, the SIRT1 activator, RES, inhibits activator protein-1 (AP-1) transcriptional activity by up-regulating SIRT1 expression levels, further inhibiting T cell activation and maintaining peripheral T cell tolerance (151). Simultaneously, RES can also induce the deacetylation of Fas, Bcl-2, Bax, and p53 by activating SIRT1 and SIRT3, and trigger Caspase 9-mediated cell apoptosis (152, 153). This mechanism achieves an inhibitory effect on CD4+ T cells and B cells, leading to a reduction in the production of anti-nuclear antibodies and the suppression of SLE immune inflammation-mediated damage to systemic tissues (154).

In 2010, Dorrie and colleagues discovered that antigen-presenting cells (APCs) present antigens to initial T cells to initiate immune responses during the recognition phase of the immune response, and some APCs present antigens to differentiated T cells during the effector phase to trigger antigen elimination. RES can influence the maturation of dendritic cells and their antigen-presenting ability *in vitro* (155). Additionally, RES can inhibit COX expression by suppressing NF-κB activation and inhibit TNF-α-induced inflammation response in fibroblasts by exciting SIRT1 (156), thereby reducing the deposition of immune complexes in the kidneys (157). Therefore, inflammation inhibition mediated by RES may also be beneficial in reducing the occurrence of lupus nephritis. In their investigation of lupus nephritis, Miyake et al. delineated the pivotal roles of Th1 and Th17 cells in the pathogenesis of diffuse proliferative lupus nephritis, while emphasizing the significance of Th2 cell cytokines in membranous lupus nephritis (158). Subsequently, Wang et al. utilized RES as a therapeutic intervention and elucidated its dose-dependent reduction in the Th1/Th2 cell ratio, thereby ameliorating the deleterious effects associated with diffuse proliferative lupus nephritis (159). Moreover, this study yielded a groundbreaking discovery, demonstrating that RES effectively mitigates proteinuria, diminishes IgG and IgM deposition in renal tissues, and attenuates renal histopathological lesions. These findings collectively suggest that RES holds promise in halting the progression of lupus nephritis.

Neuropsychiatric lupus erythematosus (NPSLE) is mainly characterized by brain vasculitis mediated by vascular endothelial growth factor (VEGF) (160). The polymorphism of genes encoding VEGF is closely related to the increased incidence of neuropsychiatric lupus (161). In 2018, Kalinowska-Lyszczarz et al. treated lupus-prone atherosclerotic mice with RES and found a significant decrease in VEGF expression, indicating that RES can reduce the occurrence of NPSLE inflammation, promote neuronal survival, and improve memory (162). The latest research indicates that any trends observed in RES-treated NPSLE mice are A2A receptor-dependent (163). Moreover, in microglia, RES counteracts the negative effects of A2A receptors by exciting SIRT1, reducing the expression of fractalkine (CX3CL1), inhibiting inflammatory responses, and improving cognitive function (164). In conclusion, the anti-inflammatory properties of RES can exert strong protective effects in NPSLE.

#### 5.1.3 Systemic sclerosis (SSc)

SSc is a chronic autoimmune connective tissue disease characterized by fibrosis and vascular abnormalities of the skin and internal organs. The disease usually severely affects the skin, oesophagus, lungs, heart, kidneys, and other organ systems, and manifests itself in a diverse range of clinical symptoms and modes of disease progression (165).The onset of SSc is geographically and ethnically diverse. The incidence of SSc is notably higher in regions such as Europe, North America, and Australia, while relatively low rates are observed in Asia, Africa, and Latin America. Additionally, the prevalence of SSc is lower among individuals of African descent and Asians, whereas those of European descent and Indian Americans exhibit higher rates. Typically, SSc affects individuals in the young to middle-aged demographic, spanning ages 20 to 50. Factors contributing to its onset include infections, occupational exposures, substance use, and lifestyle choices (166). The risks associated with SSc are multifaceted. Firstly, there’s the potential for damage to both the skin and internal organs. SSc induces fibrosis and dysfunction in these areas, where skin sclerosis can significantly impact appearance and quality of life, while organ involvement may result in compromised function, such as pulmonary fibrosis, cardiac issues, and renal impairment. Secondly, vascular complications are common. SSc often presents with both microangiopathy and macrovascular lesions, potentially leading to symptoms like Raynaud’s phenomenon and hypertension. Thirdly, there’s immune system dysregulation to consider. SSc is characterized by autoimmune processes, wherein aberrant immune activation can spur the production of autoantibodies and inflammatory cytokines, precipitating tissue inflammation and damage. Lastly, joint and muscle involvement can occur, manifesting as arthritis, joint swelling, and muscle weakness, which can significantly hinder daily activities and motor function (167).

Research suggests that the decline in SIRTs levels and activity contributes to the pathogenesis of SSc (168). Conversely, activating SIRTs can induce anti-fibrotic effects, offering therapeutic benefits for SSc patients. Specifically, when SIRT1 is activated, it inhibits the TGF-b/SMAD signaling pathway, reducing collagen release by fibroblasts and blocking tissue fibrosis (169). Notably, Zhu and colleagues discovered that RES inhibits inflammatory factor expression by activating the SIRT1/mTOR signaling pathway. By downregulating mTOR, it diminishes collagen levels, alleviating skin inflammation and fibrosis in SSc (170). Additionally, RES enhances SIRT3 expression, increases intracellular TGF-b deacetylation, hinders intracellular TGF-b signaling and fibrotic responses, and mitigates the activated phenotype of fibroblasts in SSc (171). This leads to a substantial reduction in mitochondrial and cytoplasmic ROS accumulation in fibroblasts, lessening oxidative stress damage (172). In conclusion, RES improves fibrosis by activating SIRT1 and SIRT3, thereby inhibiting TGF-b-induced signal transduction. Moreover, RES activates SIRT1 to obstruct the mTOR pathway, restraining collagen fiber accumulation. Consequently, RES emerges as a potential pharmaceutical intervention for treating SSc.

### 5.2 Organ-specific autoimmune diseases

#### 5.2.1 Graves disease (GD)

GD arises from the immune system erroneously attacking thyroid tissue, leading to hyperactivity of the thyroid gland and excessive secretion of thyroid hormones, causing an elevated metabolic rate. Approximately one-third of patients may also develop eye conditions, such as protruding eyes and eyelid swelling, known as Graves’ ophthalmopathy (GO) (173). GD, a prevalent autoimmune disease globally, predominantly affects women aged 20–40, with genetics and environmental factors closely linked to its onset. Particularly, environmental factors such as smoking, excessive iodine intake, and ionizing radiation play significant roles (174). Hazards associated with GD encompass thyroid dysfunction, thyroid storm, eye complications, an increased risk of cardiovascular diseases, and osteoporosis (175–178).

Kim et al. illustrated that RES inhibits adipogenesis in orbital fibroblasts associated with GO (179). Acting as a broad-spectrum SIRTs activator, RES diminishes the rate of ROS generation triggered by oxidative stress and lowers heme oxygenase-1 (HO-1) levels through the activation of SIRT1/3. This concurrent reduction extends to superoxide dismutase Cu/Zn-SOD (SOD1), catalase, and thioredoxin (Trx), thereby reducing adipocyte numbers and lipid droplet accumulation. Furthermore, RES induces adipocyte apoptosis within fibroblasts (180, 181). Consequently, RES holds promise in mitigating orbital lipid deposition, ameliorating ocular complications of GO, and emerging as a significant therapeutic modality for managing GD and its ocular sequelae. Nonetheless, the research in this domain remains limited, necessitating further investigation into RES’s therapeutic effects on GO via the SIRT1 signaling-dependent pathway.

#### 5.2.2 Type 1 diabetes mellitus (T1DM)

T1DM is a chronic metabolic disorder characterized by insufficient or complete lack of insulin production, leading to elevated blood sugar levels. This disease is typically caused by autoimmune destruction of pancreatic β-cells, rendering insulin unable to be effectively secreted (182). T1DM is more common in children, adolescents, or young adults and is associated with factors such as family history, viral infections, early-life environment, and dietary factors. It is worth noting that T1DM is associated with other autoimmune diseases (such as RA, autoimmune thyroid diseases, etc.), suggesting the presence of common genetic or immune factors (183). Its hazards mainly include several aspects: Firstly, cardiovascular diseases. Hyperglycemia increases the risk of cardiovascular diseases, including myocardial infarction, stroke, atherosclerosis, etc., which are among the main causes of death in diabetic patients (184). Secondly, retinal lesions. High blood sugar damages retinal blood vessels, leading to retinal lesions and potential blindness (185). Thirdly, renal damage. High blood sugar harms the kidneys, leading to diabetic nephropathy, which may require dialysis or kidney transplantation in severe cases (186). Fourthly, neuropathy. Hyperglycemia damages the nervous system, causing peripheral neuropathy (such as sensory abnormalities, pain) and autonomic neuropathy (such as gastrointestinal dysfunction, cardiovascular dysfunction) (187). Fifthly, foot complications. Neuropathy and vascular changes increase the risk of foot infections and ulcers, potentially leading to amputation (188).

In 2015, Côté et al.’s study showed that compared with saline, RES significantly increased the levels of NAD+ and its ratio to NADH in the duodenal mucosa (189). Subsequently, by detecting the acetylation level of liver kinase B1 (Lkb1) in cells after RES treatment, it was further confirmed that RES significantly increased SIRT1 activity (190–192). Specifically, RES activates AMPK, thereby activating SIRT1 to achieve its biological effects (192). The activation of the Glp1r-Pka dependent signaling in the duodenum is downstream of the metformin-AMPK sensory pathway (193), so the duodenum naturally becomes a site of action for RES. Therefore, RES reduces hepatic glucose production (HGP) by activating the duodenal AMPK-Sirt1→Glp1r-Pka dependent neural pathway, exerting a hypoglycemic effect similar to metformin (194). This not only lays the foundation for the development of intestine-specific targeted therapy but also collaboratively reduces HGP and blood sugar in diabetic and obese patients, becoming a new targeted treatment for diabetes.

RES is not only one of the new therapeutic drugs for lowering blood sugar but also has broad prospects in the prevention and treatment of diabetes-related complications. In 2016, Park et al. showed that RES can increase the expression of adiponectin receptors 1/2 (AdipoR 1/2) and activate the AMPK-SIRT1-PGC-1α axis, thereby alleviating endothelial dysfunction caused by lipotoxicity, oxidative stress, and apoptosis, and preventing diabetic nephropathy (195, 196). At the same time, RES can also prevent diabetes-induced nephritis and mesangial cell proliferation by inhibiting the Akt/NFκB pathway (197). Subsequent studies have found that RES reduces ROS production and lowers blood sugar by activating the AMPK pathway and inhibiting the expression of NADPH oxidase 4 (NOX 4) in renal tubular epithelial cells (198). In summary, RES reduces renal fibrosis and restores renal function by regulating the AMPK/SIRT1/NOX 4/ROS signaling pathway (199), achieving prevention and control of diabetic nephropathy, and reducing the immune-inflammatory damage of the kidneys caused by diabetes.

Negi et al. found that long-term high blood sugar levels can cause oxidative stress in the body, leading to the activation of the transcription factor NF-κB in peripheral neurons (200). However, NF-κB can mediate the release of a large number of pro-inflammatory cytokines such as iNOS, TNF-α, IL-6, and COX-2, thereby driving nerve damage mediated by neuroinflammation and causing peripheral neuropathy (201). In 2020, Huang et al. found that RES can reduce the inflammatory mediators in diabetic neuropathy (202). Specifically, RES upregulates the expression of SIRT1 and downregulates the expression of NF-κB, thereby reducing the activity of pro-inflammatory factors (such as TNF-α, IL-6, and iNOS) associated with neurodegeneration (203). Furthermore, RES can also ameliorate sensory motor disturbances associated with diabetic neuropathy by inhibiting the activity of TNF-α and nicotinamide adenine dinucleotide phosphate oxidase through activating SIRT1 (204, 205). Therefore, RES reduces the release of pro-inflammatory factors in diabetic patients, prevents oxidative stress from attacking neuronal cells, protects nerve function, and alleviates neuropathy.

*In vitro* studies have shown that RES reduces the expression of VEGF in human retinal endothelial cells and inhibits high glucose-induced cell proliferation (206). It can also inhibit the migration of retinal endothelial cells by activating the PI3K/Akt pathway mediated by matrix-derived factors (207). Therefore, RES has potential value in preventing diabetic retinal lesions.

#### 5.2.3 Inflammatory bowel disease (IBD)

IBD is a group of chronic intestinal disorders, mainly including Crohn’s disease (CD) and ulcerative colitis (UC). The hallmark of these diseases is chronic inflammation of the intestinal mucosa, often accompanied by ulcer formation (208). The incidence of IBD varies in different regions, with higher rates in North America, Europe, and Australia, and relatively lower rates in Asia, Africa, and Latin America. IBD is more common in adolescents and young adults aged 20–40 and is associated with genetic factors, lifestyle, dietary habits, and infections. Particularly, smoking is closely associated with an increased risk of IBD (209). Its hazards mainly include intestinal inflammation and ulcers, malnutrition, intestinal obstruction, intestinal fistulas, and an increased risk of colorectal cancer (210–212).

Many *in vitro* and animal studies have shown that RES can reduce the severity of intestinal inflammation in IBD. However, the beneficial effects of RES on treating IBD are attributed to multiple mechanisms, which ultimately lead to the inhibition of the inflammatory cascade response (213). Ren et al. demonstrated that RES primarily exerts its inhibitory effect on NF-κB by activating SIRT1, thus mitigating the severity of IBD (214). Specifically, RES suppresses the transcriptional activity of p65 and the ubiquitination of NF-κB essential modulator (NEMO) in a dose-dependent approach, thereby inhibiting NF-κB activation mediated by the NF-κB kinase. Singh et al. found that RES not only downregulates TH1 responses to reduce the secretion of inflammatory factors such as IL-1β, IL-6, and TNF-α in colitic mice but also reduces the percentage of CXCR3+ T cells. More importantly, RES significantly induces the expression of immunosuppressive CD11b+ Gr-1+ myeloid-derived suppressor cells (MDSCs) in the colon, which helps suppress local effector T cell responses (215). Furthermore, RES markedly diminishes the populations of inflammatory CD4+ and CD8+ T cells, B cells, NK cells, and myeloid-derived suppressor cells (MDSCs), underscoring its efficacy in reversing the advancement of chronic colitis (216).

In a 2012 study on UC, it was proposed that IL-17, IL-10, and transforming growth factor β1 (TGF-β1) participate in the development of TH17 through two important pathways: mTOR-HIF-1β-TH17 and IL-6-STAT3-HIF-1β-TH17. RES precisely regulates the balance between Treg and TH17 cells by activating the SIRT1/mTOR/HIF-1β/TH17 signaling pathway, leading to cell cycle arrest in intestinal smooth muscle cells, increased apoptosis, and decreased collagen synthesis (217). Subsequently, in a study on CD, RES effectively reduced the expression of insulin-like growth factor 1 (IGF-1) and procollagen mRNA by activating SIRT1, thereby significantly decreasing the activity of inflammatory cytokines (IL-1β, IL-6, and TNF-α) and the fibrosis-promoting factor (TGF-β1) (218). This study elucidated that RES not only mitigates the inflammatory response but also impedes the fibrotic process of the cecal wall.

Furthermore, Arslan et al. found that RES can enhance the activity of superoxide dismutase (SOD), catalase (CAT), and glutathione (GSH) *in vivo* (219). The study report by Serra showed that RES is more effective than 5-aminosalicylic acid and can activate two pathways, nuclear factor erythroid 2-related factor 2 (Nrf2) (220), and PPAR-γ in human intestinal cells (221, 222). It is worth noting that these two pathways are currently considered as new therapeutic targets for IBD.

In summary, RES can inhibit ROS in the intestines and increase the activity of antioxidant enzymes through multiple pathways, thereby suppressing oxidative stress to maintain the balance between oxidants and antioxidants in the body, to some extent reducing the activity of IBD and improving the quality of life of patients with IBD.

#### 5.2.4 Pulmonary fibrosis (PF)

PF is a chronic progressive lung disease and a component of connective tissue disorders. Its hallmark is the excessive proliferation and deposition of fibrous tissue in the lung tissue, leading to irreversible lung damage. This fibrosis can damage the alveoli and lung interstitium, making the lungs stiff and losing elasticity, thereby affecting respiratory function (223). PF has a relatively low incidence but is increasing annually, occurring more frequently in adult males over 60 years of age, and is associated with prolonged exposure to harmful chemicals (such as silica dust, asbestos, silicates, etc.) and inhalation of toxic gases (such as nitrogen oxides, iron oxide dust, etc.) (224). Its hazards mainly include the following aspects: firstly, impaired respiratory function. PF leads to excessive fibrous tissue proliferation in the alveoli and lung interstitium, thereby affecting lung function. Impaired lung function can cause symptoms such as dyspnea, shortness of breath, and in severe cases, may even require respiratory assistance from a ventilator, severely affecting the patient’s quality of life (225). Secondly, cardiovascular complications. PF can lead to the occurrence of cardiovascular complications such as pulmonary arterial hypertension and right heart failure, which can be life-threatening in severe cases (226). Thirdly, increased risk of cancer. Long-term chronic inflammation and tissue damage in the lungs may increase the risk of developing lung cancer (227).

In 2016, Li et al. discovered that the SIRT1 agonist RES reduces systemic oxidative stress by activating the Nrf2-related antioxidant defense mechanism, thus exerting a protective effect on PF (228). Importantly, Nrf2 can induce various downstream signaling targets, including NAD(P)H quinone dehydrogenase 1 (HO-1/NQO1), NADPH oxidase 4 (NOX4), and GSH. These signaling targets participate in the formation of multiple signaling pathways, such as the SIRT1-Nrf2-HO-1 pathway, thereby achieving anti-fibrotic effects both *in vivo* and *in vitro* through mechanisms such as inhibiting immune-inflammatory responses and oxidative stress, which significantly attenuates PF (229).The latest research indicates that RES can alleviate PF by inhibiting TGF-β-activated kinase 1 (TAK1) (230). TAK1 is a kinase involved in TGF-β activation, participating in fibroblast proliferation, collagen deposition, and scar formation (231). Zhang et al. demonstrated that moderate concentrations of RES can activate SIRT1, leading to enhanced expression of AMPK, reducing the activity of inflammatory cytokines such as IL-1β and macrophage inflammatory protein-1α (mip-1α), thereby preventing nitric oxide (NO) release, inhibiting iNOS expression, and suppressing NF-κB nuclear translocation (232). In studies on human alveolar epithelial cells (AEC2), RES upregulates the expression of SIRT1, thereby improving mitochondrial membrane potential, reducing ROS levels, decreasing cell apoptosis, ultimately reducing high oxygen-induced cell damage (233). Additionally, the activation of the SIRT1/p53 signaling pathway by RES can also delay the aging of AEC2 and participate in the treatment process of PF through its antioxidant and anti-inflammatory responses (234). Therefore, the SIRT1 and Nrf2 pathways induced by RES are likely to become potential therapeutic targets for PF.

## 6 The challenges faced in the clinical application of resveratrol and its derivatives

In recent years, there has been a surge in attention toward resveratrol and its derivatives as activators of the SIRTs family, following the discovery of their roles in metabolic regulation, cell apoptosis, cancer prevention, and other cellular processes. These compounds are recognized for their potential clinical benefits, including antioxidative, anti-inflammatory, anti-cancer, and cardiovascular protective properties. However, despite their broad range of potential applications, the clinical development of RES is hindered by its remarkably low bioavailability and susceptibility to oxidative degradation in biological formulations, thereby impeding the attainment of doses conducive to human health (106). Consequently, there is a critical need to explore various types of resveratrol derivatives to broaden and enhance the therapeutic potential of compounds resembling resveratrol, representing an urgent research imperative.

### 6.1 The severe inadequacy in bioavailability

The most pressing issue currently regarding the clinical application of RES is its bioavailability. RES’s bioavailability in the human body is affected by gradually increasing doses and repeated administration, with an oral absorption rate of approximately 75%, primarily through epithelial diffusion. However, extensive metabolism predominantly occurs in the intestines and liver, resulting in an oral bioavailability far below 1%. Even with gradual dose increases and repeated administration, this situation remains largely unchanged (65). In mammals, RES is chiefly metabolized in the intestines and liver, primarily converting to glucuronides and sulfates (235, 236). Nonetheless, its metabolism is exceedingly rapid, with plasma concentrations of RES diminishing rapidly within 30–60 minutes post-administration, posing challenges for systemic medication (237). A comparative study by Ndiaye investigated three RES administration models—drinking water, oral feeding, and sustained-release pellets implanted near tumors (238). Surprisingly, RES exhibited no effect on melanoma in the body (239), with plasma RES levels remaining very low and rapidly converting to its metabolites such as resveratrol glucoside and resveratrol. Similarly, RES application in various areas such as lung cancer, intestinal tumors, and leukemia has shown markedly low bioavailability (240, 241). Efforts to address this issue are underway. For instance, a study by Niles and others in 2003 demonstrated that leveraging RES’s characteristics via epithelial diffusion for skin cancer treatment not only tackled its rapid metabolism but also significantly enhanced its bioavailability (242). However, enhancing RES’s bioavailability remains a critical concern in the medical community.

### 6.2 The poor biological stability

RES, a bioactive polyphenol, is widely present in various foods. Despite its strong beneficial effects on human health, such as antioxidant, anti-aging, cardioprotective, neuroprotective, and chemopreventive properties (243), its use as a drug is still greatly limited by its poor bioavailability, and the tendency for auto-oxidation and photosensitivity further restricts the development of its pharmaceutical formulations (244). The instability of RES is attributed to its deprotonation in alkaline media and subsequent auto-oxidation, degradation, or polymerization processes. RES exists in cis and trans isomeric forms, with the trans isomer being more biologically active and widely used as SIRTs activators (245). However, the isomerization of trans-resveratrol is influenced by various factors such as exposure time and wavelength, physical state of the molecule, temperature, and pH (64). Research by Goldberg et al. suggests that trans-resveratrol is unstable under light and heat; when exposed to ultraviolet radiation for several hours, trans-resveratrol can convert to cis-resveratrol, significantly losing its biological stability (246). Additionally, Robinson et al. investigated the stability of both resveratrol isomers in aqueous solutions and the kinetic effects of this process (247). The results indicate that under light protection, trans-resveratrol can remain stable for at least 42 hours in neutral buffer and at least 28 days in acidic medium. Therefore, the urgent need to address the issues of RES stability in terms of photo and water stability is crucial to maintain its beneficial effects, prevent the formation of oxidation or photo-oxidation products, and avoid harm from its metabolites to human health. Recent evidence suggests that the limitations of RES stability and bioavailability can be circumvented by developing new formulations or synthesizing new prodrugs (248). Studies by Dhakar et al. found that RES formulated into nanosponge encapsulated systems achieved higher water solubility and drug loading than single drugs. Furthermore, nanosponges dissolve drugs in a controlled manner, significantly prolonging the release duration of RES. Moreover, the antioxidant activity of RES is further enhanced in the presence of nanosponges. Most importantly, nanosponges exhibit biocompatibility and do not cause significant cytotoxicity to the organism. Therefore, future research should focus on the development of new products to make RES more soluble and delay its metabolism.

### 6.3 The dosage range and safety assessment

In addition to low bioavailability, determining the appropriate dosage range of RES and assessing the long-term safety of its use are also important challenges. In 2010, Brown et al. reported toxicity data from a dose-escalation study describing the intake of RES by 44 healthy volunteers (249). No serious adverse events were detected through clinical, biochemical, or hematological parameters, with the most common toxicity being gastrointestinal reactions. According to the Common Terminology Criteria for Adverse Events (CTCAE) from the National Cancer Institute, approximately 90% of reported events were classified as mild. However, most adverse reactions occurred in populations taking the two highest doses or individuals ingesting more than 1 gram of RES per day. Therefore, for future clinical development of RES, intake for one month at doses as high as 1 gram per day is safe and well tolerated (250). It is important to note that the concentration of RES varies in different tissues and organs. For example, there is a high concentration of RES in colonic tissue, far exceeding the concentrations required for *in vitro* activity, but the concentration in other tissues may be much lower than the appropriate *in vitro* concentration (251). Therefore, the clinical efficacy of RES likely depends to a large extent on whether its metabolites have significant activity or can regenerate RES (252). However, some studies have found different results, such as low concentrations of RES exhibiting biological activity and displaying a biphasic dose-response relationship (253). Further research is needed to explore the relationship between RES dosage and safety.

### 6.4 Adverse reactions

#### 6.4.1 Cell toxicity

Bolton et al. found that the metabolites of RES have cytoprotective effects but can also induce cytotoxicity or immunotoxicity (254). During the metabolism of RES, cytochrome P450 generates quinone compounds through hydroxylation reactions, producing the metabolite piceatannol. Piceatannol possesses anti-inflammatory and antioxidant properties. It not only enhances the expression of the antioxidant enzyme heme oxygenase-1 (HO-1) in human breast epithelial cells by inducing Nrf2 (255), but also inhibits the downregulation of the anti-apoptotic B-cell lymphoma 2 protein (Bcl-2), thereby suppressing oxidative stress and apoptosis induced by hydrogen peroxide and peroxynitrite (256). The quinone metabolites of RES are also associated with toxic effects, involving oxidative stress and alkylation mechanisms (257). For example, piceatannol can induce the inhibition of P450 oxidase or alkylation of certain proteins such as Keap1, Nrf2, I kappa B kinase (IKK), where NF-κB can lead to severe hepatorenal toxicity (258). Moreover, quinone compounds can deplete GSH, affect the function of nicotinamide adenine dinucleotide phosphate oxidase (NOX), and ultimately lead to oxidative stress reactions, damaging normal organisms (254). Studies have shown that tyrosinase, a key enzyme involved in melanin biosynthesis, participates in RES metabolism. RES can serve as a substrate for tyrosinase, generating active quinone compounds (259). The generated quinone compounds can decay to form oligomers, which as pro-oxidants, can trigger cytotoxicity in melanocytes, ultimately leading to the development of skin tumors (260).

#### 6.4.2 DNA damage

Research has shown that increasing intake of RES can better clear ROS, thus, RES has a cellular protective effect (259). However, under certain conditions, antioxidants may also act as pro-oxidants, leading to accelerated lipid peroxidation or induced DNA damage (261). In fact, whether RES exhibits pro-oxidant or antioxidant activity depends on RES concentration, form, processing conditions, and its redox status (262, 263). When acting as a pro-oxidant molecule *in vitro*, RES can cause DNA damage, reduce multiple DNA repair pathways, and activate cytotoxicity and apoptosis pathways (264). Zuo et al. found that RES exhibits preferential cytotoxicity in malignant tumor cells, leading to higher electron transfer between RES and copper ions (265). Therefore, RES and copper-induced DNA damage may be one of the cytotoxic mechanisms of RES against cancer cells (266). It is noteworthy that the pro-oxidative action of RES has pro-apoptotic function in different types of cancer cells, and its ability to induce DNA breaks has potential therapeutic value in combating cancer cells (267). However, there are also reports that RES triggers p53-dependent apoptosis by activating topoisomerase II, thereby inducing DNA damage in colon cancer cells (268). In U2OS and A549 cancer cells treated with RES, the proportion of DNA double-strand breaks significantly increased. This phenomenon may also be mediated by RES-induced pro-oxidative effects and regulation of the CXCR2-p53 pathway (269).

#### 6.4.3 Induction of oxidative stress

Giordo et al. found that RES severely impacts cellular redox state (270). In this regard, low doses of RES have various beneficial effects, such as protecting cells and tissues from the effects of neurodegeneration, cardiovascular diseases, cancer, diabetes, and obesity-related diseases, and prolonging organism lifespan (271). However, there is substantial evidence indicating that RES exhibits a biphasic concentration-dependent effect. That is, both *in vivo* and *in vitro*, RES acts as an antioxidant at low doses and a pro-oxidant at high doses (272). The pro-oxidative effect of RES is typically associated with the downregulation of phosphorylated pkb/Akt, cell damage, and apoptosis. Meanwhile, ROS-mediated mitochondrial damage produced by cytochrome P450 enzyme cyp2c9 in high doses of RES-induced oxidative damage seems to be involved as well (273). Heo et al. proposed that RES can induce apoptosis and cell cycle arrest in malignant melanoma cells (274). Additionally, Kim found that RES induces caspase-dependent cell death in ovarian cancer cells through a ROS-dependent mechanism. It is worth mentioning that in their 2022 study, Elbaz et al. found that resveratrol also triggers the downregulation of miR-144 by upregulating the expression level of Nrf2.Consequently, resveratrol showed hepatorenal antioxidant, anti-inflammatory, and antiapoptotic activities as manifested by improvement in the antioxidant markers along with a decline in NF-κB, TNF-α, and caspase-3 expressions. In summary, this study demonstrated that resveratrol has potential therapeutic effects in alleviating liver and kidney damage induced by nonsteroidal anti-inflammatory drugs (275).

#### 6.4.4 Drug interactions

Currently, an increasing number of studies have found indirect interactions between RES and other drugs, leading to decreased activity or overexpression of major cellular systems involved in drug metabolism—drug transporters and CYP450 enzyme activity (276). Previous studies have shown that RES can blunt the function and expression of drug transporters, thereby increasing the anti-proliferative activity of various drugs and reducing their bioavailability (277). For example, RES inhibits drug transport proteins such as P-glycoprotein, multidrug resistance-associated protein 2 (MRP2), and organic anion transport proteins (OAT1/OAT3), reducing the clearance of methotrexate in the kidneys and increasing the risk of liver toxicity (278, 279). Additionally, RES can enhance the anticoagulant activity of warfarin, increasing the risk of bleeding (280). Interestingly, combination therapy with RES has also been reported to attenuate the effects of other drugs. For instance, RES can diminish the effects of human immunodeficiency virus (HIV) protease inhibitors (281) and interact with inhibitors of 3-hydroxy-3-methylglutaryl-coenzyme A reductase (HMG-CoA reductase) (282), immunosuppressants (283), antiarrhythmic drugs (284), antihistamines (285), and calcium channel agonists (286), thereby reducing the bioactivity and utilization of drugs and affecting drug efficacy.

### 6.5 The turning point between basic research and clinical practice

In addition, the majority of research on RES is currently still in the basic research stage, and transitioning it into clinical practice promptly poses a significant challenge. The lag in clinical application is due to RES being a natural compound with many clinically relevant targets, which have different dose-response curves, tissue distributions, and modifiers. Effectively addressing these issues is currently a challenge. New paradigms and methods need to be developed, including better molecular modeling to predict interactions; large-scale screening for toxicity or other common effects; and affinity-based methods to identify drug interactions, among others (287).

## 7 Conclusion and outlook

This study systematically explores the mechanism and potential applications of resveratrol and its derivatives in the treatment of AID. Through literature review, we found that RES, as a SIRTs activator, has significant therapeutic potential in regulating immune cell function and suppressing the release of inflammatory factors, providing new insights for the treatment of AID.

Despite some encouraging progress, several challenges persist and must be addressed. These challenges encompass the bioavailability, stability, and safety of resveratrol and its derivatives. Future research endeavors should focus on, but not be restricted to, the following key areas: firstly, delving deeper into assessing the efficacy and safety of resveratrol and its derivatives across various types of AID. Secondly, enhancing the bioavailability, stability, and specificity of resveratrol and its derivatives through advancements in synthesis, drug design, and targeted delivery systems. Thirdly, formulating personalized treatment plans tailored to individual genetic, phenotypic, and lifestyle factors to achieve the precision medicine goal, considering the potential variation in the impact of resveratrol and its derivatives among individuals. Lastly, exploring the molecular mechanisms of the SIRTs pathway in immune regulation and devising more selective and specific SIRTs modulators to mitigate adverse reactions and side effects.

In conclusion, resveratrol, as a potential AID treatment, holds expansive application prospects; however, further research and clinical validation are imperative. Collaborative efforts within the medical community are essential to propel the advancement of this field, offering patients more efficacious and safer treatment alternatives.

## Author contributions

XY: Data curation, Funding acquisition, Investigation, Methodology, Writing – original draft. MC: Investigation, Resources, Visualization, Writing – review & editing. JW: Investigation, Resources, Validation, Writing – review & editing. RS: Conceptualization, Funding acquisition, Resources, Validation, Writing – review & editing.
